# Recovery expectations of neck pain patients do not predict treatments outcome in manual therapy

**DOI:** 10.1038/s41598-020-74962-5

**Published:** 2020-10-28

**Authors:** J.-H. A. M. Mutsaers, A. L. Pool-Goudzwaard, R. Peters, B. W. Koes, A. P. Verhagen

**Affiliations:** 1 Institute for Master Education in Manual Therapy, SOMT, Amersfoort, The Netherlands; 2 Department of General Practice, Erasmus MC, University Medical Centre Rotterdam, P.O. Box 2040, 3000 CA Rotterdam, The Netherlands; 3 Avans Hogeschool, University of Applied Sciences, P.O. Box 90116, 4800 RA Breda, The Netherlands; 4 Research Institute MOVE, Faculty of Human Movement Sciences, VU University Amsterdam, Van der Boechorststraat, 9, 1081 BT Amsterdam, The Netherlands; 5 Center for Muscle and Joint Health, University of Southern Denmark, Odense, Denmark; 6 Discipline of Physiotherapy, Graduate School of Health, University of Technology Sydney, Sydney, Australia

**Keywords:** Pain management, Rehabilitation, Outcomes research, Musculoskeletal system

## Abstract

Patient recovery expectations can predict treatment outcome. Little is known about the association of patient recovery expectations on treatment outcome in patients with neck pain consulting a manual therapist. This study evaluates the predictive value of recovery expectations in neck pain patients consulting manual therapists in the Netherlands. The primary outcome measure ‘recovery’ is defined as ‘reduction in pain and perceived improvement’. A prospective cohort study a total of 1195 neck pain patients. Patients completed the Patient Expectancies List (PEL) at baseline (3 item questionnaire, score range from 3 to 12), functional status (NDI), the Global Perceived Effect (GPE) for recovery (7-points Likert scale) post treatment and pain scores (NRS) at baseline and post treatment. The relationship between recovery expectancy and recovery (dichotomized GPE scores) was assessed by logistic regression analysis. Patients generally reported high recovery expectations on all three questions of the PEL (mean sumscores ranging from 11.3 to 11.6). When adjusted for covariates the PEL sum-score did not predict recovery (explained variance was 0.10 for the total PEL). Separately, the first question of the PEL showed predictive potential (OR 3.7; 95%CI 0.19–73.74) for recovery, but failed to reach statistical significance. In this study patient recovery expectations did not predict treatment outcome. Variables predicting recovery were recurrence and duration of pain. The precise relationship between patient recovery expectations and outcome is complex and still inconclusive. Research on patient expectancy would benefit from more consistent use of theoretical expectancy and outcome models.

## Introduction

Patient recovery expectations are defined as patient’s perceptions that a certain outcome of medical care is likely to occur^1,2^. Among other factors, for medical care, personal experiences and those of family members and acquaintances develop these recovery expectations. Recovery expectations can also be influenced by the interactions that a patient has with the healthcare provider^3^.

Recovery expectancies are believed to influence treatment outcome through mechanisms that are still largely unknown. One of the theoretical frameworks that can help unravel these mechanisms is the response expectancy theory^4^. This theory encompasses two relevant aspects of medical treatment: the patient as a passive recipient of treatment and the patient’s volitional health-directed behavior. The first aspect refers to the expected occurrence of the individual’s non-volitional, internal responses to a certain external stimulus (e.g., the expectation that an analgesic will lead to pain reduction). The second aspect refers to the outcome expectancies of one’s own volitional health-directed behavior (e.g., the expectation that a relaxation exercise will reduce subjective stress). Patient recovery expectations have the potential to influence treatment adherence and outcome. So far expectancy research within the realm of physical and manual therapy is limited and mainly aimed at low back pain. The results vary, with some studies failing to find predictive value for patient expectancy^5,6^, and others succeeding in doing so^7–11^.

A recent study on neck pain patients pre-treatment expectations were found to be related to patients’ ratings of recovery at 1- and 6-months post treatment (exercise and manipulation)^12^. At 1 month, patients with lower expectation on pain relief had a lower chance of recovery than those with high expectancies on pain relief (OR 0.33, 95% CI 0.11; 0.99). The expectation that spinal manipulation would help while not receiving it also lowered the chance of treatment success (OR 0.16, 95% CI 0.04; 0.72) compared to expecting spinal manipulation and actually receiving it. Similar results were found for the influence of expectation on functional status in this study^12^.

Neck pain is a common musculoskeletal disorder with an estimated point prevalence of 9–22% in the general population of the Netherlands^13^. Approximately one third of all adults is likely to experience neck pain during the course of 1 year^14^. Neck pain patients often seek help from manual therapists. Current guidelines incorporate known prognostic factors, but assessing expectancy prior to treatment is not a guideline recommendation. A deeper understanding of the influence of recovery expectancy on the treatment outcome in patients with neck pain consulting a manual therapist, could help improve guidelines, clinical decision making and patient outcome. This study aims to evaluate the predictive value of recovery expectancy of neck pain patients on outcome for manual therapy in the Netherlands.

## Methods

### Study design

This study is part of a large prospective cohort study with 12 months follow up in a Dutch manual therapy setting studying the associations between pain attitudes, treatment choices and outcome expectations of manual therapists and non-specific neck pain patients. For this study only demographic data and data on expectancy, functional status and recovery post treatment were extracted from the database.

### Participants

#### Manual therapists

The manual therapists (n = 272) included in this study took part in a part-time 3-year course, aimed to reregister certified Dutch manual therapists with an internationally recognised Master of Science degree. All participating manual therapists were asked to include five consecutive patients of 18 years and over within a time frame of 6 months, that consulted them for their neck pain. Each new patient was immediately recorded in the database, providing insight in the inclusion flow.

#### Patients

All adult patients consulting with non-specific neck pain were eligible. Neck pain is defined as pain located in the area between occiput and the spinae scapulae^15^. Excluded were all patients with known specific causes of neck pain (e.g. known vascular or neurological disorders, neoplasms, rheumatic conditions, referred pain from internal organs) and patients who were unable to read and/or write Dutch. The patients received information on the study and signed an informed consent to be included in the study. Demographic information (i.e., gender, age) was collected through the participating manual therapists at baseline, including those individuals who were screened for eligibility, qualified for the study, but refused to participate. This information was only used to check representativeness of the study group. Ethical approval for this study was obtained from the Medical Ethical Committee (MEC-2007-359) from Erasmus University Rotterdam, the Netherlands.

### Baseline measurement

#### Manual therapists

Socio-demographic and professional data were collected and comprised gender, age, occupational setting, number of hours at work, number of years of experience with the management of non-specific neck pain patients.

#### Patients

Baseline data (age, gender, type of complaint, recurrence, duration of complaints) were recorded and all patients completed a baseline questionnaire including the Numeric Rating Scale (NRS) for pain intensity, functional status (Neck Disability Index (NDI), and the Patient Expectations List (PEL). The PEL is based on the two aspects of the response expectancy theory and was developed by expert consensus specifically for use in a Dutch manual therapy setting. It models ‘expectancy’ as a two-component variable consisting of ‘treatment modality’ and ‘conviction’. It consists of 3 questions, each with a sub-question (see Box 1). Each question generates a combined score with the sub-question, varying from ‘1’ (low expectation and strongly convinced), ‘2’ (low expectation and not strongly convinced), ‘3’ (high expectation and not strongly convinced) to “4” (high expectation and strongly convinced). PEL sum scores for the three questions are generated and range from 3 to 12. We added an extra dichotomous question that checks earlier experiences with manual therapy (yes/no), which will be analyzed as a confounder.

**Box 1:** The patient expectancies list (PEL).

1	To what extent do you expect your neck pain to change as a result of the overall therapeutic approach?
1a	To what extent are you convinced that this will be the case?
2	To what extent do you expect your neck pain to change as a result of spinal manipulation?
2a	To what extent are you convinced that this will be the case?
3	To what extent do you expect your neck pain to change as a result of exercise?
3a	To what extent are you convinced that this will be the case?

Since the clinimetric properties of none of the separate PEL-questions (PEL-1, PEL-2 or PEL-3) have been evaluated so far, we will analyse the separate questions as well as the PEL sum scores.

### Post treatment measurement

At the end of the individual treatment episodes, pain and the primary outcome ‘recovery’ were assessed with the Numeric pain rating scale (NRS) and Global Perceived Effect (GPE) respectively. On the GPE scale the patient scored on a 7-points Likert scale how much their condition improved or deteriorated since the start of the treatment, ranging from ‘complete recovery’ to ‘worse than ever’. The GPE has several qualities that make it an appealing tool for use in clinical practice and research; being a single question, it is easy and quick to administer and the results are seemingly simple to interpret^16,17^. The GPE was reassessed at 12 months.

### Analyses

We used descriptive statistics (SPSS version 20.0) to summarize the baseline and post-treatment data. The independent variable was patient expectation. PEL scores were calculated for separate questions as well as for the total PEL and with the exception of the added dichotomous question, analysed as continuous variables. PEL scores for acute and non-acute neck pain patients were calculated separately. The outcome of interest was recovery (measured using the GPE) post treatment. The recovery data post treatment and at 12-months follow-up were dichotomized into “recovered” (scores ‘completely recovered’ and ‘much improved’) and “not recovered” (‘slightly improved’ to ‘worse than ever’). Recovery data at 12 months follow-up were compared to the post treatment data for stability of recovery with McNemar’s test.

As possible confounders patient age, gender, functional status, baseline pain scores, duration and recurrence of neck pain, smoking, and sports participation were entered in the analyses^18,19^.

Concerning missing data, first we evaluated whether there are specific patterns of missing data using Little’s MCAR test. We also compared baseline data between patients with and without missing data. In case this test was negative we performed multiple imputation to overcome a loss of power due to missing’s. Both predictor and outcome variables were included in the multiple imputation^19,20^. A total of 10 datasets were created and analysis was performed on all datasets. Pooled estimates were calculated according to Ruben’s rules^21^. All candidate predictors derived from the literature were checked for multicollinearity. Association between candidate variables and recovery was checked using Chi-square tests. Correlation coefficients ≤ 0.35 were considered to represent low association, 0.36–0.67 modest correlations, and 0.68–0.89 high and ≥ 0.90 very high correlations^22,23^.

Univariate analyses were performed on single PEL questions and PEL sum scores separately. Next the univariate analysis were adjusted for previous experiences with manual therapy as a possible confounder to evaluate the association of expectancy and recovery.

Lastly, we performed a multivariate analysis (using Backward Wald) to build a prognostic model. To be able to adhere to the criterion of at least 10 events per variable we selected the variables with a *p* < 0.10 in the univariate analysis^24^. Overall performance of the model will be expressed by Nagelkerke’s R^2^ and the discriminant ability using the area under the curve (AUC). An AUC of 1.0 indicates perfect discrimination, between 0.8 and 1 indicates acceptable discrimination, between 0.7 and 0.8 fair discrimination, whereas an AUC of 0.5–0.7 indicates poor discrimination above chance^25^. The goodness-of-fit of the model was determined with the Hosmer–Lemeshow statistic^26^.

## Results

### Participants

#### Manual therapists

The majority (79%) of the manual therapists (MPTs) were male, with a mean age of 42.2 (SD 8.4) years, a work experience of 19.3 (SD 7.1) years, averaging almost 24.6 (SD 10.2) hours of work per week in a general practice, with a mean weekly number of neck pain patients of 12.2 (SD 8).

#### Patients

Post-treatment data were available for 663 (50.5%) patients, 1 year follow-up data for 385 (29.4%). The demographic data of the patients are presented in Table 1. A total of 1311 patients (62.8% female) was enrolled, with a mean age of 44.7 years. Most of them reported recurrent (66.9%) and/or non-acute (> 6 weeks duration) neck pain with concomitant symptoms, most frequently consisting of headache (31.1%) and irradiating arm pain (21%). Within the study population, 456 (34.8%) patients had earlier treatment experience with the manual therapist for their musculoskeletal conditions and 49.7% consulted the manual therapists through direct access. The participants are similar to the group of non-responders and non-participants concerning age and gender. From the eligible non-participants (n = 2618), 63.2% was female, with a mean age of 44.9 (SD 16.6).

**Table 1:** Patient characteristics at baseline.

Variable	All participants (n = 1311)		Recovered* (n = 523)		Not recovered* (n = 140 )	
Gender; female (%)	62.8%		79%		64.1%	
Age in years; mean (SD)	44.7 (13.7)		46.1 (13.7)		48.3 (13.8)	
Recurrent neck pain (%)	66.9%		68.7%		57.5%	
**Referral**						
Direct access	49.7%		64%		36%	
General practitioner	44.7%		58.3%		41.7%	
Other	6.6%					
Marital status; married (%)	76.9%		74.4%		68.3%	
Work status; employed (%)	77.1%		72.9%		65.8%	
Smoking (%)	25.2%		27.3%		22.5%	
Practising sports (%)	65.9%		61.5%		60.8%	
Concomitant symptoms (%)	20.7%		19.3%		21.5%	
NRS pain (n = 1183), mean (SD)	4.8 (2.1)		4.8 (2.0)		4.9 (2.1)	
NDI sumscore, mean (SD)	26.5 (6.5)		22.8 (6.4)		23.2 (5.8)	
Earlier treatment experience (%)	34.8%		36.2%		31.6%	
Duration of neck pain (n)	< 6 weeks 512	> 6 weeks 799	< 6 weeks 234	> 6 weeks 289	< 6 weeks 34	> 6 weeks 106
PEL-1: mean (SD); median	3.9 (0.2); 4.0	3.9 (0.4); 4.0	3.9 (0.1); 4.0	3.9 (0.3); 4.0	3.9 (0.2; 4.0)	3.8 (0.5); 4.0
PEL-2: mean (SD); median	3.9 (0.3); 4.0	3.8 (0.5); 4.0	3.9 (0.3); 4.0	3.8 (0.5); 4.0	3.9 (0.2); 4.0	3.8 (0.4); 4.0
PEL-3 : mean (SD); median	3.7 (0.7); 4.0	3.5 (1.0); 4.0	3.7 (0.8); 4.0	3.6 (0.8); 4.0	3.8 (0.3); 4.0	3.5 (0.9); 4.0
PEL-sumscore _:_ mean (SD); median	11.6 (1.0); 12.0	11.3 (1.4); 12.0	11.7 (1.2); 12.0	11.4 (1.2); 12.0	11.8 (0.6); 12.0	11.3 (1.3); 12.0

*Available post treatment GPE scores; NRS, Numeric Rating Scale; NDI, Neck Disability Index; PEL, Patient Expectancies List.

### Recovery expectation and treatment outcome

**Figure 1:**
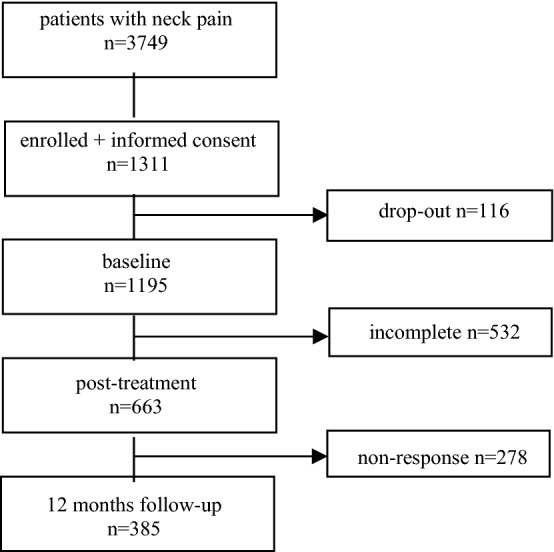
Flow chart of the study.

Patients reported high recovery expectations. Overall 31% of the respondents stated that their recovery expectations were partly based on earlier positive experiences with manual therapy. Due to non-response, complete data on recovery post-treatment were available for 663 patients, of which 523 patients (79%) were classified as ‘recovered’ (see Fig. 1) after receiving a mean number of treatments of 5.4 (SD 2.6). At 12 months follow-up, data were available for 385 patients, of which 303 reported to be ‘recovered’.

Sum-scores were generally high (85.7% scored > 9, range 3–12). Scores for question 1 and 2 yielded slightly higher recovery expectations (means 3.9 (SD 0.4 and 0.5 resp.), range 1–4) than recovery expectations for question 3 (mean 3.6 (SD 0.9), range 1–4). All PEL scores showed negative Skewness and Kurtosis. No differences were found in the total PEL scores, or the individual PEL items, between acute and non-acute neck pain patients. Mc Nemar’s test showed that there were no differences in recovery between the post-treatment measurement and the 12-month follow-up, suggesting that recovery was stable (Table 2).

**Table 2:** GPE post treatment and at 12 month follow-up (Mc Nemar’s test).

n	Mean (SD)			
582	0.79 (0.40)	GPE post treatment	Not recovered	Recovered
385	0.79 (0.41)	**GPE follow-up**		
		Not recovered	73	1
		Recovered	1	286

*GPE* Global Perceived Effect.

### Prediction and modelling

A multi collinearity check revealed that no variables had to be withheld from the analysis because of high correlation.

#### Univariate analysis

Unadjusted ORs for the separate questions of the were 4.04 (0.56–28.98), 0.44 (0.05–3.49) and 0.97 (0.94–1.32) for PEL-1, PEL-2 and PEL-3 respectively (Table 3). When adjusted for earlier experience with manual therapy, the OR (95%CI) for PEL-1 increased slightly to 4.79 (0.51–45.20). When we adjust for all other possible confounders the analysis revealed similar results for the separate PEL questions, with OR’s ranging from 3.73 (0.19–73.74) for PEL-1, to 0.19 (0.01–2.82) for PEL-2 (Table 4). The OR’s for the covariates all performed poorly, except for ‘duration of pain’, with OR’s of 3.43 (1.955–6.00) and 3.17 (0.75–2.16) for acute (less than 6 weeks) and non-acute (more than 6 weeks) respectively. When considering a predictive model, the analyses yielded results in which only ‘duration of pain’, acute and non-acute were represented as positive predictors and in which ‘patient recovery expectations’ do not contribute.

**Table 3:** Univariate regression.

	Univariate, raw	Adjusted for earlier experience
	OR [95% CI]	OR [95% CI]
PEL1	4.0 [0.6–28.9]	4.8 [0.5–45.2]
PEL2	0.4 [0.1–3.5]	0.4 [0.1–3.6]
PEL3	0.9 [0.5–1.9]	0.9 [0.5–1.9]
PEL-total	1.1 [0.9–1.3]	

*PEL* Patient Expectancies List.

**Table 4:** Multivariate regression for separate expectation scores predicting treatment outcome.

	Range	Multivariate (enter)	Predictive model
		OR [95% CI]	OR [95% CI]
PEL1	1–4	3.7 [0.2–73.7]	
PEL2	1–4	0.2 [0.1–2.8]	
PEL3	1–4	0.7 [0.3–1.6]	
NDI	0–42	0.9 [0.9–1.0]	
NRS	1–10	0.9 [0.8–1.2]	
Gender		0.9 [0.5–1.8]	
Age	18–83	0.9 [0.9–1.0]	
Duration < 6 weeks		3.4 [1.9–6.0]	3.3 [1.9–6.0]
Duration > 6 weeks		3.1 [1.4–7.3]	2.6 [1.2–5.9]
Recurrent		1.3 [0.7–2.2]	
Medication		0.9 [0.5–1.7]	
Smoking		1.1 [0.6–1.8]	
Sports		0.7 [0.4–1.2]	

Performance measures of the model			
AUC (95%CI)		Correctly classified (%)	R^2^
0.68 (0.65–0.74)		80.8	0.104

*PEL*, Patient Expectancies List, *NDI*, Neck Disability Index, *NRS* Numeric Rating Scale.

Analyses for PEL sum scores yielded similar results (Table 5), with slightly lower ORs for ‘duration of pain’ (acute, 3.28 (1.87–5.75); non-acute, 2.96 (1.28–6.85)) in the predictive model.

**Table 5:** Multivariate regression for summed expectation scores predicting treatment outcome.

	Predictive model	
	OR [95% CI]	OR [95% CI]
PEL-total	1.0 [0.8–1.2]	
NDI	0.9 [0.9–1.0]	
NRS	0.9 [0.8–1.1]	
Gender	0.9 [0.5–1.6]	
Age	0.9 [0.9–1.0]	
Acute	3.3 [1.8–5.7]	3.4 [1.9–6.0]
Subacute	2.9 [1.2–6.8]	2.6 [1.2–5.9]
Recurrent	1.3 [0.7–2.2]	
Medication	0.9 [0.5–1.7]	
Smoking	1.1 [0.6–1.9]	
Sports	0.7 [0.4–1.2]	

Performance measures of the model		
AUC (95%CI)	Correctly classified (%)	R^2^
0.68 (0.64–0.70)	80.5	0.094

*PEL* Patient Expectancies List, *NDI* Neck Disability Index, *NRS* Numeric Rating Scale.

#### Model performance

The explained variance (R^2^) of the final models was 0.9 (9%) and 0.10 for separate questions and total PEL respectively. This means that the models explain 9 to 10 percent of recovery. The ROC curve of the model for the total PEL showed a relatively poor discriminating ability for the model with a AUC of 0.675 (0.65–0.74). The models correctly predicted recovery for 80% of the patients.

## Discussion

### Main findings

In this study patients had a high and seemingly stable overall recovery rate and high overall recovery expectations of therapy. We found that patient recovery expectations of separate questions of the PEL, as well as the overall PEL score, did not predict treatment outcome. In this study only ‘shorter duration of pain’ and ‘first episode of neck pain’ were positive predictors of recovery.

### Comparison with literature

Although about half of the population reported not to have based their recovery expectations on earlier experiences with manual therapy, they still expected that spinal manipulation would bring them favourable results (PEL 2). Closely followed by the belief that exercise therapy would add to treatment effects (PEL 3). These high recovery expectations seem to be in line with research done on outcome expectancy in other fields of healthcare^27,28^.

In this study we found that recovery expectations as measured by the PEL did not predict outcome in terms of recovery. These inconclusive results on the predictive value of expectancy are in line with earlier research. Studies that do find predictive value of patient expectancies on recovery^12,28^, are countered by studies that fail to establish predictive value of patient expectancies on recovery^29,30^.

One of the factors that may contribute to these inconsistent findings, is the heterogeneity of the conceptualization and assessment of patients’ recovery expectations^31^. Some studies use different terminology for overlapping qualities of expectancy^32^, others highlight only one or several aspects of expectancy^33^. Without a more uniform and detailed insight in the make-up of expectancy, comparison and integration of current findings is compromised. Based on perspectives from several human sciences, Thompson and Sunol proposed a helpful distinction between four types of expectation: ideal, predicted, normative and unformed. Their exact make-up and relation to terms such as ‘hope’ and ‘satisfaction’ are yet to be disentangled^34^.

The effects of terminology may reach even further in expectancy research. Questions on spinal manipulation and exercise assume a uniform definition and meaning of what they consist of and what they can do. That assumption may very well be flawed. How reliably can one quantify expectancy if there is insufficient or at least varying insight in what ‘spinal manipulation’ and ‘exercise’ exactly are? It can be hypothesised that other studies in this field are influenced by the same mechanism, contributing to contrasting results. Lastly, the application of the GPE may add to inconsistent findings. The underlying assumptions of the GPE is that it measures a composite of multiple domains relative to ‘improvement’ or ‘recovery’ of one’s condition, but knowledge on the factors patients take into account when determining their GPE, is still limited. A mixed-method study on de GPE revealed five main themes patients used to construct ‘recovery’, and that chronic neck pain patients have different expectations of recovery than non-chronic neck pain patients. Not expecting to fully recover, may lead to reponse-shift. Lastly the GPE seems to be strongly affected by ‘current status’ instead of ‘stable change’ especially as the transition time lengthens^35^.

In this study the covariates ‘initial pain intensity’ and ‘functional limitation’ did not contribute to the predictive model. Results of earlier research suggest that high baseline neck pain intensity and high functional limitation have a strong association with outcome^34,35^. Although pain scores in this study seem typical for the population^35^, the limited variance and low numbers of ‘non-recovery’ may have been the reason for lacking association. Although the majority of patients in this study reported longer existing neck pain, only 31% had earlier experience with manual therapy. The level of evidence for manual therapy is moderate for short-term effects of upper thoracic manipulation in acute neck pain, limited for long-term effects of neck manipulation, and limited for all techniques and follow-up durations in chronic neck pain^35–37^. Research on prognostic factors of neck pain has shown that a vast number of predictors provide low predictive value or inconclusive results, suggesting there is still much work to be done in this field^35,38^. We non-directionally postulated that earlier experience may influence outcome expectation by providing either ‘lived reference’ to the patient that may be a dominant factor in the make-up of their expectancy.

### Strength and limitations

This study explored the predictive value of patient recovery expectations on outcome for manual therapy in a large group of patients. It contributes to the growing insight in the associations between patient expectancy and treatment outcome in general, and incorporates manual therapy and non-specific neck pain in the scope of research in this field.

A limitation of this study is number of missings in recovery scores, the high dropout rate and the limited variance (e.g. most patients reported relatively high PEL scores at baseline) in recovery expectancy, all negatively impacting the statistical possibilities to detect differences and associations. Some form of selection bias cannot be ruled out. Therapists were asked to include five consecutive patients and apart from inclusion flow and cross comparison with non-enrolled patients there was no process installed that guaranteed adherence to the inclusion process. Furthermore, measuring recovery expectancy as a nearly single factor variable seems to be an oversimplification of reality, blurring opportunities to find out more about its make-up and in- and external dynamics. For instance, it is unclear to what degree patients reported ‘ideal’ expectancy based on motivation instead of ‘predicted’ expectancy based on cognition and/or earlier experience. Since patient expectations on recovery were under-modelled and overall high in this study, and the GPE was dichotomised, the capacity to find possible associations with recovery may have been limited.

Another limiting factor in this study is the developmental stage of the PEL. The PEL is a newly developed questionnaire and insight in its psychometric properties is lacking. There is limited insight into its psychometric properties.

### Implications

For practice. Understanding patient recovery expectations of treatment outcome is an important part of developing treatment plans and stimulating therapy adherence. Even though evidence is still sparse and inconclusive, there still may be practical reasons to measure recovery expectations . It may be a quick and reproducible route to an ‘agreement on treatment’ with your patients, since it provides possibilities to ‘synchronise’ preferences on treatment modalities and expectations of their outcome.

For research. More collaboration is needed on adopting an integrative model of expectancy that incorporates aspects of the common sense model, process or structural recovery expectations and the valence of patients’ recovery expectations^31^. Analogously, adopting a more frequent and nuanced GPE measurement that differentiates between acute and non-acute patients, would improve the capacity to detect possible associations between expectancy and outcome. It would also be relevant to focus on the dynamics and influenceability of recovery expectations during treatment, their physiological make-up and possible capability to influence favourable outcome^39^.

## Conclusions

Patient recovery expectations did not predict treatment outcome in this study. Variables predicting recovery were recurrence and duration of pain. Research on patient expectancy would benefit from more consistent use of theoretical expectancy models.

## Ethical approval

All methods used in this study were carried out in accordance with relevant clinical guidelines and regulations on manual therapy for non-specific neck pain.
